# Association between pre-stroke anemia and risk of acute kidney injury following ischemic stroke: A multi-institutional retrospective cohort study

**DOI:** 10.1097/MD.0000000000051449

**Published:** 2026-11-06

**Authors:** I-Wen Chen, Chia-Li Kao, Yi-Chen Lai, Kuo-Chuan Hung

**Affiliations:** a Department of Anesthesiology, Chi Mei Hospital, Liouying, Tainan, Taiwan; b Department of Anesthesiology, E-Da Hospital, I-Shou University, Kaohsiung, Taiwan; c Department of Anesthesiology, Chi Mei Medical Center, Tainan, Taiwan; d School of Medicine, College of Medicine, National Sun Yat-sen University, Kaohsiung, Taiwan.

**Keywords:** acute kidney injury, anemia, hemoglobin, prognosis, propensity score matching, stroke

## Abstract

Although anemia is an established risk factor for acute kidney injury (AKI), its specific impact on post-stroke AKI remains inadequately explored. This study aimed to investigate the association between pre-stroke anemia and the risk of developing AKI after acute ischemic stroke. This retrospective study, using the TriNetX research network database, identified adult patients who experienced their 1st recorded ischemic stroke between January 2010 and December 2023. Patients were categorized based on hemoglobin levels within 3 months pre-stroke: anemia (hemoglobin ≥8.0 and <12.0 g/dL) versus control (hemoglobin ≥12.0 g/dL). After 1:1 propensity score matching, we analyzed the outcomes at 1-year follow-up. The primary endpoint was the development of AKI during the follow-up period. Secondary endpoints comprised all-cause mortality, pneumonia, and intensive care unit admission. After propensity score matching, 61,896 patients were included in each cohort. Patients with pre-stroke anemia had a significantly higher 1-year risk of AKI (hazard ratio [HR]: 1.89, 95% confidence interval [CI]: 1.83–1.96), mortality (HR: 1.86, 95% CI: 1.80–1.93), pneumonia (HR: 1.76, 95% CI: 1.69–1.84), and intensive care unit admission (HR: 1.60, 95% CI: 1.55–1.65) compared to controls (all *P* < .001). A graded association was observed between increasing anemia severity and adverse outcomes. The findings remained robust across sensitivity and subgroup analyses. Pre-stroke anemia is associated with an increased risk of post-stroke AKI and other adverse outcomes. However, given the observational nature of this study, future prospective studies are warranted to confirm these findings and elucidate the underlying mechanisms.

## 1. Introduction

Stroke remains a leading cause of mortality and disability worldwide. In 2019, there were approximately 12.2 million new stroke cases worldwide, comprising 62.4% ischemic strokes (around 7.63 million cases), 27.9% intracerebral hemorrhages (approximately 3.41 million cases), and 9.7% subarachnoid hemorrhages (about 1.18 million cases).^[1]^ Among stroke survivors, acute kidney injury (AKI) has emerged as a frequent complication, affecting 11.6% to 20.9% of patients following an acute stroke.^[2–6]^ This post-stroke complication significantly impacts clinical outcomes, with studies demonstrating that patients who develop AKI after stroke face a 2- to 3-fold higher risk of in-hospital mortality,^[6–8]^ prolonged hospital stays, and increased risk of chronic kidney disease (CKD) development.^[9,10]^ Furthermore, the development of AKI following acute ischemic stroke was independently associated with poorer functional recovery at 3 months, with 88.1% of patients with AKI exhibiting moderate to severe disability compared to 51.3% of those without AKI.^[11]^ Identifying risk factors for post-stroke AKI is therefore of paramount importance, as this may enable clinicians to implement targeted preventive strategies, optimize management protocols, and potentially mitigate the adverse consequences of this complication on stroke recovery and long-term outcomes.

Anemia has been well-established as a risk factor for AKI in various clinical settings. In surgical populations, preoperative anemia or low hemoglobin was associated with an increased risk of AKI in patients undergoing cardiac and noncardiac surgery.^[12–15]^ Evidence from nonsurgical patient cohorts suggests that anemia present at hospital admission is an independent risk factor for AKI.^[16]^ In a retrospective study of over 34,000 hospitalized individuals, those with anemia demonstrated a significantly higher incidence of AKI (11.2%) compared to their non-anemic counterparts (5.5%), even after adjusting for potential confounders.^[16]^ Similarly, in a cohort of critically ill patients admitted to the intensive care unit (ICU), anemia was consistently linked to an elevated risk of AKI, irrespective of its onset or duration.^[17]^ Despite these established relationships in various populations, the specific impact of pre-stroke anemia on post-stroke AKI remains inadequately explored. Based on these observations across diverse clinical scenarios, we hypothesize that pre-stroke anemia increases the risk of post-stroke AKI. This study aimed to examine the relationship between pre-stroke anemia and the risk of developing AKI after acute stroke during both short- and long-term follow-up.

## 2. Methods

### 2.1. Data source and ethical statement

We conducted a retrospective, multi-institutional study using the TriNetX (TriNetX, LLC) research network database. The TriNetX Research Network is a federated, de-identified electronic health record database that aggregates data from a diverse range of academic medical centers, tertiary referral hospitals, and community healthcare systems, predominantly located in the United States, with additional international contributors.^[18]^ This database has been widely used and cited in numerous peer-reviewed clinical and epidemiological studies.^[19–21]^ This study was conducted in accordance with the Declaration of Helsinki and received approval from the Institutional Review Board of Chi Mei Medical Center (IRB no. 11403-E01), which waived the requirement for informed consent owing to the use of de-identified data.

### 2.2. Patient selection and inclusion criteria

We identified adult patients (aged ≥18 years) who experienced their 1st recorded ischemic stroke between January 2010 and December 2023. Ischemic stroke was identified using the International Classification of Diseases, 10th revision, Clinical Modification (ICD-10-CM) code I63, which encompasses both acute and subacute ischemic stroke. The 1st recorded ischemic stroke was defined as the earliest recorded diagnosis of ischemic stroke (International Classification of Diseases, 10th revision, Clinical Modification code I63) in the TriNetX database, and the date of this diagnosis served as the index date. To ensure data quality and continuity of care, we included only patients with at least 2 visits to the same healthcare organization. Patients were categorized based on their hemoglobin levels within 3 months prior to the stroke event: the anemia group (hemoglobin ≥8.0 and <12.0 g/dL) and the control group (hemoglobin ≥12.0 g/dL), regardless of sex.^[22]^

TriNetX does not currently support sex-specific aggregation of laboratory values for cohort classification; consequently, anemia status was defined using a uniform hemoglobin threshold across sexes, with patients classified as anemic (hemoglobin ≥8.0 and <12.0 g/dL) or control (hemoglobin ≥12.0 g/dL) based on this single set of criteria. For patients with multiple hemoglobin measurements within the 3-month pre-stroke window, the timing of individual measurements was not weighted according to proximity to the index stroke. All available measurements within this period were considered, and patients were classified as anemic or control only when their hemoglobin values met the corresponding prespecified group criteria; patients with values outside the prespecified range were excluded to minimize exposure misclassification.

### 2.3. Exclusion criteria

We excluded patients with: hemoglobin <8 g/dL; intracerebral hemorrhage within 1 year before or after ischemic stroke; history of cystic kidney disease, end-stage renal disease, or systemic lupus erythematosus; history of pneumonia, ICU admission, coronavirus disease 2019, AKI, or intracranial injury within 3 months prior to acute ischemic stroke; development of contrast-induced AKI within 2 weeks after acute ischemic stroke; and history of surgery or hemorrhagic events within 2 weeks prior to acute ischemic stroke. The exclusion criteria were designed to minimize confounding and reverse causation.

### 2.4. Data collection and propensity score matching

We extracted demographics (age, sex, and race), health status factors, comorbidities (e.g., hypertension, diabetes, and heart disease), laboratory values (albumin, hemoglobin A1c [HbA1c], and estimated glomerular filtration rate [eGFR]), body mass index, and medication use (e.g., angiotensin-converting enzyme inhibitors, sodium–glucose cotransporter 2 inhibitors, and nonsteroidal anti-inflammatory drugs) from the TriNetX database. Information regarding fasting status at the time of laboratory testing was not available in the TriNetX dataset. No imputation was performed for missing laboratory values. The availability and corresponding missingness of albumin, HbA1c, and eGFR in the propensity score-matched cohorts are reported in Table S1, Supplemental Digital Content 1. Information on blood transfusion before or shortly after stroke was not available and therefore could not be included as a covariate. To balance the baseline characteristics between the anemia and control groups, we performed propensity score matching using variables including demographics, comorbidities, laboratory values, and medications. Patients were matched in a 1:1 ratio using a nearest-neighbor algorithm with a caliper width of 0.1 standard deviations of the logit of the propensity score.

### 2.5. Outcomes

The primary outcome was the 1st recorded AKI event during the follow-up period, identified using diagnostic coding available in the TriNetX database. Serum creatinine- and urine output-based KDIGO criteria were not used for outcome ascertainment. Recurrent AKI episodes during follow-up were not analyzed as separate events. Patients with AKI within 3 months before the index stroke were excluded, whereas a more remote history of AKI was not an exclusion criterion. The secondary outcomes included all-cause mortality, pneumonia, and ICU admission. All outcomes were assessed over a 1-year follow-up period from the index date of ischemic stroke. Recurrent ischemic strokes occurring during follow-up were not treated as censoring events. To evaluate the impact of anemia on short-term outcomes, we examined the same outcomes at 30 days post-stroke.

### 2.6. Sensitivity analysis

We conducted 3 sensitivity analyses to evaluate the robustness of our findings. For model I, the analysis was restricted to patients who experienced stroke between 2010 and 2018. This timeframe was selected to avoid the confounding effects of the coronavirus disease 2019 pandemic. For model II, the analysis was restricted to patients treated at medical centers. For model III, analysis was limited to patients who survived for at least 1 year after stroke. This survivor-restricted analysis was performed as a sensitivity analysis for potential competing mortality, particularly in relation to longer-term renal complications. It was not considered a formal competing-risk analysis.

### 2.7. Subgroup analysis

Two predefined subgroup analyses were performed. First, we categorized the anemia group into 2 mutually exclusive categories: mild anemia (hemoglobin ≥10.0 and <12.0 g/dL) and severe anemia (hemoglobin ≥8.0 and <10.0 g/dL). Each category was compared separately with the control group (hemoglobin ≥12.0 g/dL). This analysis aimed to assess whether a graded association was observed between increasing anemia severity and adverse outcomes. Second, we conducted a sex-specific analysis to assess whether the impact of anemia on post-stroke outcomes differed between male and female patients. To address the potential misclassification associated with the uniform threshold used in the primary analysis, the male-specific analysis defined anemia as hemoglobin 8 to 12.9 g/dL and the control group as hemoglobin ≥13 g/dL.

### 2.8. Association between pre-stroke anemia and 30-day outcomes

To evaluate the immediate impact of pre-stroke anemia on early post-stroke outcomes, we analyzed the association between anemia status and all primary and secondary outcomes within the 1st 30 days of the index stroke event. This analysis provides insights into the acute effects of anemia on post-stroke complications and can guide early interventions in the management of stroke patients with anemia.

### 2.9. Statistical analysis

All statistical analyses were performed using the TriNetX platform. Data are presented as mean with standard deviation or number with proportion. All outcome data are presented as hazard ratios (HR) with 95% confidence intervals (CI). In the primary analysis, HRs were estimated using Cox proportional hazards models, treating death as a censoring event, thereby reflecting cause-specific associations between pre-stroke anemia and the risk of AKI among patients remaining at risk. The proportional hazards assumption was assessed for each outcome using the test for proportionality provided as part of the TriNetX survival analysis. As the TriNetX analytic framework does not support formal competing-risk modeling, such as Fine–Gray subdistribution hazards or cumulative incidence functions, the survivor-restricted model III analysis was interpreted as a sensitivity analysis for potential competing mortality rather than as a formal competing-risk analysis. Group balance was assessed via standardized mean differences (SMDs), with values <0.1 denoting sufficient equivalence. To address multiplicity, we prespecified a single primary outcome and several secondary outcomes. Statistical significance for the primary outcome was defined as a 2-sided *P* value <.05. Secondary outcomes were considered exploratory in nature; therefore, no formal adjustment for multiple comparisons was applied to these analyses. Accordingly, the possibility of false-positive findings among the secondary endpoints cannot be excluded, and these findings should be interpreted as hypothesis-generating.

## 3. Results

### 3.1. Patient selection and baseline patient characteristics

As illustrated in Figure 1, we initially identified patients with a 1st recorded ischemic stroke in the TriNetX database. After applying our inclusion and exclusion criteria, 62,751 patients were assigned to the anemia group and 254,954 patients to the control group. Prior to propensity score matching, patients in the anemia group were older (71.3 ± 15.1 vs 66.7 ± 14.8 years), more likely to be female (63.6% vs 44.2%), and had a higher prevalence of comorbidities including diabetes mellitus, ischemic heart disease, heart failure, and CKD (i.e., stage 3 and stage 4). After 1:1 propensity score matching, 61,896 patients remained in each group. The matching process successfully balanced baseline characteristics between groups, as evidenced by an SMD of <0.1 for all variables. In the matched cohorts, the mean age was 71.2 ± 15.2 years in the anemia group and 71.6 ± 14.2 years in the control group (SMD = 0.028) (Table 1).

**Table 1:** Baseline characteristics of patients with a 1st recorded ischemic stroke before and after propensity score matching.

Variables	Before matching			After matching			
	Anemia group (n = 62,751)	Control group (n = 254,954)	SMD*		Anemia group (n = 61,896)	Control group (n = 61,896)	SMD*
Patient characteristics							
Age at index (yr)	71.3 ± 15.1	66.7 ± 14.8	0.303		71.2 ± 15.2	71.6 ± 14.2	0.028
BMI <18.5 (kg/m^2^)	1861 (3.0%)	3529 (1.4%)	0.109		1722 (2.8%)	1661 (2.7%)	0.006
BMI 30–40 (kg/m^2^)	8740 (13.9%)	36,629 (14.4%)	0.013		8513 (13.8%)	8171 (13.2%)	0.016
Female	39,912 (63.6%)	112,700 (44.2%)	0.397		39,217 (63.4%)	39,608 (64.0%)	0.013
White	26,919 (42.9%)	125,122 (49.1%)	0.124		26,682 (43.1%)	27,119 (43.8%)	0.014
Factors influencing health status and contact with health services	25,751 (41.0%)	92,367 (36.2%)	0.099		25,033 (40.4%)	24,794 (40.1%)	0.008
Comorbidities/medication							
Essential (primary) hypertension	21,194 (33.8%)	74,571 (29.2%)	0.098		20,529 (33.2%)	20,362 (32.9%)	0.006
Diabetes mellitus	13,191 (21.0%)	36,483 (14.3%)	0.177		12,575 (20.3%)	12,222 (19.7%)	0.014
Ischemic heart diseases	10,173 (16.2%)	30,406 (11.9%)	0.123		9704 (15.7%)	9489 (15.3%)	0.010
Neoplasms	9593 (15.3%)	28,135 (11.0%)	0.126		9266 (15.0%)	9350 (15.1%)	0.004
Heart failure	6715 (10.7%)	14,069 (5.5%)	0.191		6219 (10.0%)	6118 (9.9%)	0.005
Overweight and obesity	4222 (6.7%)	15,062 (5.9%)	0.034		4048 (6.5%)	3804 (6.1%)	0.016
COPD	3858 (6.1%)	11,671 (4.6%)	0.070		3690 (6.0%)	3572 (5.8%)	0.008
Nicotine dependence	3162 (5.0%)	15,930 (6.2%)	0.052		3104 (5.0%)	3002 (4.9%)	0.008
Diseases of liver	2313 (3.7%)	7082 (2.8%)	0.051		2223 (3.6%)	2138 (3.5%)	0.007
Malnutrition	1222 (1.9%)	1560 (0.6%)	0.119		1044 (1.7%)	993 (1.6%)	0.006
COVID-19	1060 (1.7%)	3628 (1.4%)	0.022		1008 (1.6%)	970 (1.6%)	0.005
Alcohol related disorders	936 (1.5%)	3912 (1.5%)	0.004		917 (1.5%)	895 (1.4%)	0.003
Chronic kidney disease, stage 1	143 (0.2%)	327 (0.1%)	0.024		134 (0.2%)	127 (0.2%)	0.002
Chronic kidney disease, stage 2	768 (1.2%)	1828 (0.7%)	0.052		712 (1.2%)	728 (1.2%)	0.002
Chronic kidney disease, stage 3	4479 (7.1%)	8006 (3.1%)	0.182		4026 (6.5%)	3826 (6.2%)	0.013
Chronic kidney disease, stage 4	1729 (2.8%)	1216 (0.5%)	0.181		1209 (2.0%)	1016 (1.6%)	0.023
Chronic kidney disease, stage 5	399 (0.6%)	126 (0.0%)	0.100		211 (0.3%)	118 (0.2%)	0.029
Long term use of NSAID	557 (0.9%)	1737 (0.7%)	0.023		541 (0.9%)	574 (0.9%)	0.006
Laboratory data							
Albumin g/dL (≥3.5 g/dL)	21,099 (33.6%)	85,177 (33.4%)	0.005		20,549 (33.2%)	20,449 (33.0%)	0.003
Hemoglobin A1c (≥7%)	5677 (9.0%)	18,088 (7.1%)	0.072		5411 (8.7%)	5162 (8.3%)	0.014
eGFR (≥60 mL/min/1.73 m^2^)	16,456 (26.2%)	75,133 (29.5%)	0.072		16,296 (26.3%)	16,268 (26.3%)	0.001
Medications							
Antilipemic agents	16,078 (25.6%)	55,724 (21.9%)	0.089		15,493 (25.0%)	15,240 (24.6%)	0.009
ACE inhibitors	7927 (12.6%)	28,193 (11.1%)	0.049		7708 (12.5%)	7600 (12.3%)	0.005
Angiotensin II inhibitor	7884 (12.6%)	23,890 (9.4%)	0.102		7539 (12.2%)	7440 (12.0%)	0.005
Covid-19 vaccines	2650 (4.2%)	10,265 (4.0%)	0.010		2569 (4.2%)	2500 (4.0%)	0.006
SGLT2 inhibitors	884 (1.4%)	4066 (1.6%)	0.015		865 (1.4%)	845 (1.4%)	0.003

ACE = angiotensin-converting enzyme, BMI = body mass index, COPD = chronic obstructive pulmonary disease, COVID-19 = coronavirus disease 2019, eGFR = estimated glomerular filtration rate, NSAID = nonsteroidal anti-inflammatory drug, SGLT2 = sodium–glucose cotransporter 2, SMD = standardized mean difference.

* SMD values <0.10 were considered to indicate adequate balance after propensity score matching.

**Figure 1.**
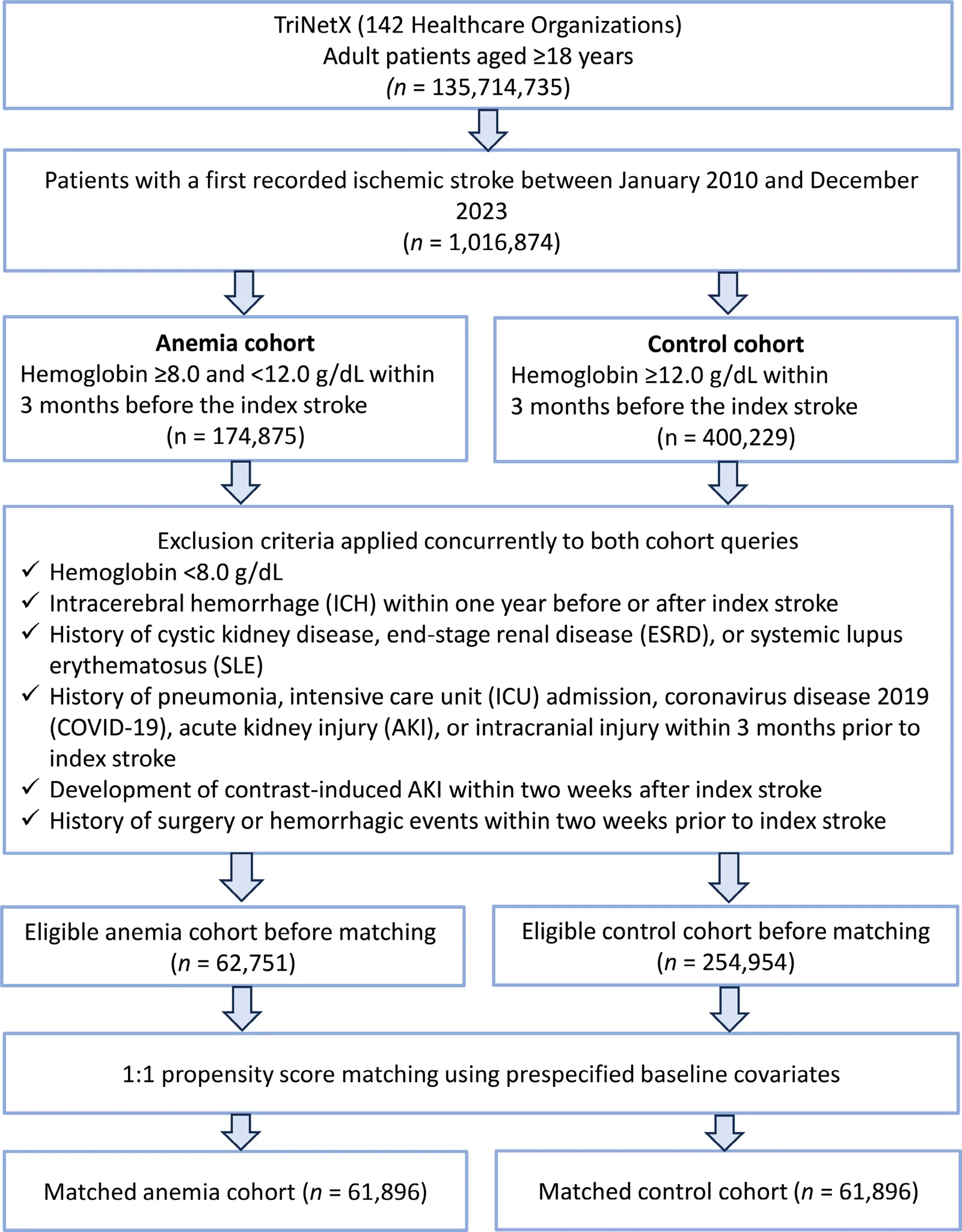
Patient selection and cohort construction from the TriNetX database. Inclusion and exclusion criteria were applied concurrently within the cohort queries and could overlap within individual patients; therefore, criterion-specific sequential exclusion counts are not presented. The figure shows the final eligible cohort sizes before and after propensity score matching.

### 3.2. Association between pre-stroke anemia and 1-year outcomes

During the 1-year follow-up period, patients with pre-stroke anemia demonstrated a significantly higher risk of adverse outcomes compared to the matched control group (Table 2). The incidence of AKI was substantially higher in the anemia group than the control group (14.6% vs 8.5%, HR 1.89, 95% CI 1.83–1.96, *P* < .001). The absolute 1-year risk difference for AKI was 6.1 percentage points (95% CI 5.7–6.4), corresponding to an approximate number needed to harm of 17. All-cause mortality was also markedly elevated in patients with anemia (14.5% vs 8.4%, HR 1.86, 95% CI 1.80–1.93, *P* < .001). Other secondary outcomes showed similar patterns, with the anemia group experiencing higher risks of pneumonia (HR 1.76, 95% CI 1.69–1.84, *P* < .001) and ICU admission (HR 1.60, 95% CI 1.55–1.65, *P* < .001). The proportionality tests were nonsignificant for all-cause mortality (*P* = .159) and ICU admission (*P* = .189), whereas significant departures from proportional hazards were observed for AKI and pneumonia (both *P* < .001). Therefore, the corresponding Cox hazard ratios should be interpreted as summary estimates over the 1-year follow-up period rather than as constant relative hazards over time.

**Table 2:** Association between pre-stroke anemia and 1-year outcomes.

Outcomes	Anemia group (n = 61,896)	Control group (n = 61,896)	HR (95% CI)	*P* value	Absolute risk difference, %	Approximate NNH
	events (%)	events (%)				
Acute kidney injury	9046 (14.6%)	5287 (8.5%)	1.89 (1.83–1.96)	<.001	6.1	17
All-cause mortality	8954 (14.5%)	5193 (8.4%)	1.86 (1.80–1.93)	<.001	6.1	17
Pneumonia	5256 (8.5%)	3263 (5.3%)	1.76 (1.69–1.84)	<.001	3.2	32
ICU admission	9500 (15.3%)	6272 (10.1%)	1.60 (1.55–1.65)	<.001	5.2	20

Absolute risk difference represents the difference in observed 1-year risks between the anemia and control groups. The approximate number needed to harm (NNH) was calculated as the reciprocal of the absolute risk difference and rounded up to the nearest whole number. Given the observational study design, NNH is presented solely as a descriptive measure of absolute risk and should not be interpreted causally.

CI = confidence interval, HR = hazard ratio, ICU = intensive care unit.

### 3.3. Sensitivity analyses of the association between pre-stroke anemia and 1-year outcomes

All 3 sensitivity analyses corroborated our primary findings, demonstrating the robustness of the association between pre-stroke anemia and adverse outcomes (Table 3). In model I, which included only patients who experienced stroke between 2010 and 2018 (n = 37,822 per group), anemia remained significantly associated with increased risks of AKI (HR 1.84, *P* < .001), mortality (HR 1.82, *P* < .001), pneumonia (HR 1.67, *P* < .001), and ICU admission (HR 1.51, *P* < .001).

**Table 3:** Sensitivity analyses of the association between pre-stroke anemia and 1-year outcomes.

Outcomes	Model I		Model II		Model III	
	HR (95% CI)	*P* value	HR (95% CI)	*P* value	HR (95% CI)	*P* value
Acute kidney injury	1.84 (1.76–1.92)	<.001	1.88 (1.81–1.96)	<.001	1.91 (1.83–1.99)	<.001
All-cause mortality	1.82 (1.75–1.91)	<.001	1.91 (1.84–1.99)	<.001	–*	–*
Pneumonia	1.67 (1.58–1.76)	<.001	1.74 (1.64–1.83)	<.001	1.69 (1.61–1.79)	<.001
ICU admission	1.51 (1.45–1.58)	<.001	1.63 (1.57–1.69)	<.001	1.53 (1.48–1.59)	<.001

Model I: Analysis of patients with stroke between 2010 and 2018. (n = 37,822 per group).

Model II: Analysis of patients with stroke treated at a medical center (n = 38,357 per group).

Model III: Survivor-restricted sensitivity analysis including patients who survived for at least 1 year after stroke (n = 51,222 per group), performed to assess robustness in relation to potential competing mortality. This was not a formal competing-risk analysis. All-cause mortality was not evaluated because eligibility for model III required 1-year survival.

CI = confidence interval, HR = hazard ratio, ICU = intensive care unit.

* The HR and *P* value for all-cause mortality were not estimable in model III because eligibility for this analysis required survival for at least 1 year.

Model II, which restricted analysis to patients treated at medical centers (n = 38,357 per group), showed similar risk elevations for all outcomes. In model III, which excluded patients who died within the 1st year after stroke (n = 51,222 per group) as a sensitivity analysis for potential competing mortality, the association between anemia and adverse outcomes persisted, with HRs of 1.91 for AKI, 1.69 for pneumonia, and 1.53 for ICU admission.

### 3.4. Subgroup analysis

We conducted 2 predefined subgroup analyses to further characterize the relationship between anemia and post-stroke outcomes (Tables 4 and 5). First, we stratified the anemia group by severity, comparing patients with mild anemia (hemoglobin ≥10.0 and <12.0 g/dL, n = 43,278 per group) and severe anemia (hemoglobin ≥8.0 and <10.0 g/dL, n = 11,338 per group) separately to matched controls (Table 4). A graded association was observed between increasing anemia severity and adverse outcomes. Severe anemia was consistently associated with higher risks than mild anemia across all outcomes. For AKI, the HRs were 1.70 for mild anemia and 2.40 for severe anemia. Similarly pronounced gradients were observed for mortality, pneumonia, and ICU admission.

**Table 4:** Association between pre-stroke anemia severity and 1-year outcomes.

Outcomes	Mild anemia (n = 43,278 per group)		Severe anemia (n = 11,338 per group)	
	HR (95% CI)	*P* value	HR (95% CI)	*P* value
Acute kidney injury	1.70 (1.63–1.77)	<.001	2.40 (2.23–2.59)	<.001
All-cause mortality	1.53 (1.46–1.59)	<.001	2.72 (2.52–2.94)	<.001
Pneumonia	1.53 (1.45–1.62)	<.001	2.23 (2.02–2.46)	<.001
ICU admission	1.38 (1.33–1.44)	<.001	1.98 (1.85–2.12)	<.001

Mild anemia was defined as haemoglobin ≥10.0 and <12.0 g/dL, whereas severe anemia was defined as haemoglobin ≥8.0 and <10.0 g/dL. Each anemia category was compared separately with a matched control group defined by hemoglobin ≥12.0 g/dL.

CI = confidence interval, HR = hazard ratio, ICU = intensive care unit.

**Table 5:** Association between pre-stroke anemia and 1-year outcomes by sex.

Outcomes	Male (n = 35,907 per group)		Female (n = 36,321 per group)	
	HR (95% CI)	*P* values	HR (95% CI)	*P* values
Acute kidney injury	1.85 (1.77–1.94)	<.001	1.84 (1.75–1.93)	<.001
All-cause mortality	2.07 (1.97–2.17)	<.001	1.71 (1.63–1.80)	<.001
Pneumonia	1.78 (1.69–1.89)	<.001	1.61 (1.51–1.71)	<.001
ICU admission	1.49 (1.43–1.55)	<.001	1.51 (1.44–1.57)	<.001

For the sex-specific analyses, anemia was defined as haemoglobin ≥8.0 and <13.0 g/dL in males and ≥8.0 and <12.0 g/dL in females. The corresponding control groups were defined as haemoglobin ≥13.0 and ≥12.0 g/dL, respectively.

CI = confidence interval, HR = hazard ratio, ICU = intensive care unit.

Our gender-specific analysis demonstrated that anemia significantly increased the risk of all adverse outcomes, with similar patterns in both males and females (Table 5). In the male-specific analysis, using hemoglobin 8 to 12.9 versus ≥13 g/dL, pre-stroke anemia remained associated with an increased risk of AKI (HR 1.85). Similarly, the risk for all-cause mortality, pneumonia, and ICU admission was consistent across genders, suggesting that pre-stroke anemia is a significant risk factor for adverse outcomes regardless of gender.

### 3.5. Association between pre-stroke anemia and 30-day outcomes

To assess the short-term impact of pre-stroke anemia, we analyzed outcomes within 30 days after stroke onset (Table 6). The findings mirrored those of the 1-year analysis, with significantly higher rates of adverse events in the anemia group. The 30-day incidence of AKI was 7.1% in the anemia group compared to 3.8% in the control group (HR 1.94, *P* < .001). Early mortality was also substantially higher in patients with anemia (5.9% vs 3.3%, HR 1.80, *P* < .001). Other short-term outcomes showed similar patterns.

**Table 6:** Association between pre-stroke anemia and 30-day outcomes.

Outcomes	Anemia group (n = 58,746)	Control group (n = 58,746)	HR (95% CI)	*P* value
	Events (%)	Events (%)		
Acute kidney injury	4178 (7.1%)	2215 (3.8%)	1.94 (1.84–2.04)	<.001
All-cause mortality	3465 (5.9%)	1966 (3.3%)	1.80 (1.70–1.90)	<.001
Pneumonia	2073 (3.5%)	1154 (2.0%)	1.84 (1.71–1.97)	<.001
ICU admission	6880 (11.7%)	4381 (7.5%)	1.60 (1.54–1.66)	<.001

CI = confidence interval, HR = hazard ratio, ICU = intensive care unit.

### 3.6. Risk factors for acute kidney injury at 1-year follow-up after stroke

In a separate multivariable Cox proportional hazards model performed in the unmatched cohort (Table 7), pre-stroke anemia was associated with an increased risk of post-stroke AKI (HR 2.10, *P* < .001). This estimate differs from the HR of 1.89 in the primary analysis because the latter was derived from the propensity score-matched cohort. Among the measured covariates included in the unmatched multivariable model, anemia had the largest HR. The next most significant risk factor was CKD (HR 1.86, *P* < .001), followed by malnutrition (HR 1.54, *P* < .001) and heart failure (HR 1.52, *P* < .001). These factors were also associated with an increased risk of AKI in the multivariable model.

**Table 7:** Risk factors for acute kidney injury at 1-year follow-up after stroke.

Variables	HR (95% CI)	*P* value
Anemia vs control group	2.10 (2.05–2.16)	<.001
Chronic kidney disease (yes vs no)	1.86 (1.79–1.92)	<.001
Malnutrition (yes vs no)	1.54 (1.42–1.67)	<.001
Heart failure (yes vs no)	1.52 (1.46–1.58)	<.001
Age at index	1.01 (1.01–1.01)	<.001
Male vs female	1.34 (1.31–1.37)	<.001
Hypertension (yes vs no)	1.01 (0.98–1.04)	.487
Overweight and obesity (yes vs no)	1.28 (1.23–1.33)	<.001
Diabetes mellitus (yes vs no)	1.29 (1.25–1.33)	<.001
Nicotine dependence (yes vs no)	1.42 (1.36–1.49)	<.001
Ischemic heart diseases (yes vs no)	1.04 (1.00–1.07)	.045
Alcohol related disorders (yes vs no)	1.09 (1.00–1.18)	.042
COPD (yes vs no)	1.14 (1.09–1.19)	<.001
Diseases of liver (yes vs no)	1.08 (1.02–1.14)	.007
Long term use of NSAID (yes vs no)	1.11 (1.00–1.24)	.062

The multivariable Cox model was performed in the unmatched cohort.

CI = confidence interval, COPD = chronic obstructive pulmonary disease, HR = hazard ratio, NSAID = nonsteroidal anti-inflammatory drug.

## 4. Discussion

In this large multi-institutional retrospective study of 123,792 propensity-matched patients, we found that pre-stroke anemia was significantly associated with an increased risk of AKI, mortality, pneumonia, and ICU admission during the year following ischemic stroke. A graded association was observed between increasing anemia severity and adverse outcomes. Severe anemia (hemoglobin ≥8.0 and <10.0 g/dL) was associated with substantially higher risks than mild anemia (hemoglobin ≥10.0 and <12.0 g/dL). In the separate multivariable analysis of the unmatched cohort, pre-stroke anemia had the largest HR among the measured covariates included in the model. This finding should be interpreted within the context of the variables available for adjustment and should not be taken to indicate that anemia is the most important determinant of post-stroke AKI. The consistency of these associations across different sensitivity analyses, timeframes (30-day and 1-year), and patient subgroups strengthens the clinical significance of our findings.

Previous research has established anemia as a risk factor for AKI in various clinical settings, including cardiac surgery, critical illness, and general hospitalization.^[12–17]^ However, the specific relationship between pre-stroke anemia and post-stroke AKI has received limited attention. Most studies in the current literature investigating the impact of anemia on post-stroke complications have primarily focused on mortality rather than renal outcomes.^[22–25]^ A previous review article identified several key risk factors for AKI following stroke, including preexisting renal impairment, advanced age, greater stroke severity, congestive heart failure, and ischemic heart disease.^[8]^ Therefore, our investigation adds novel insights by comprehensively characterizing the anemia-AKI relationship in stroke patients, including the graded association across anemia-severity categories, short- versus long-term impacts, and relative importance compared to other risk factors.

Conceptually, we hypothesize that ischemic stroke may be viewed as an acute physiological stressor that provides a shared clinical context in which the adverse effects of pre-stroke anemia on renal function become more pronounced. While anemia has been associated with increased AKI risk across diverse clinical settings, acute stroke is accompanied by systemic inflammation, sympathetic activation, and hemodynamic instability, all of which may increase renal oxygen demand and compromise perfusion. In patients with preexisting anemia, reduced oxygen-carrying capacity may limit physiological reserve to buffer these acute perturbations, thereby increasing susceptibility to post-stroke AKI. These proposed mechanisms provide a biologically plausible explanation for the observed association but were not directly evaluated in the present study and should therefore be regarded as hypotheses rather than established causal mechanisms.

Our findings have several potential clinical implications. First, hemoglobin assessment should be integrated into standard stroke workups, with anemic patients identified for closer renal monitoring. The consistency of associations observed at both 30 days and 1 year supports the temporal consistency of the findings but should not be interpreted as evidence of causality because of the observational design. This pattern also suggests that a substantial proportion of adverse events may occur early after ischemic stroke, underscoring the importance of the early post-stroke period as a critical window for risk stratification and monitoring. While our observational design does not allow us to define specific intervention strategies, these findings support a care continuum that prioritizes early identification of high-risk patients and proactive monitoring rather than delayed management. Second, strategies to minimize nephrotoxic exposures (e.g., contrast media and nonsteroidal anti-inflammatory drugs) may be particularly important in patients with anemic stroke. Third, while our study could not establish whether treating anemia would reduce AKI risk, it raises the possibility that optimizing hemoglobin levels might represent a modifiable factor in post-stroke kidney protection. Future research should address whether anemia correction improves renal outcomes after stroke, determine optimal hemoglobin targets, and explore the underlying molecular mechanisms through prospective studies using biomarker analysis.

There was a significant association between pre-stroke anemia and the development of pneumonia following ischemic stroke in the current study. Post-stroke pneumonia is a well-established complication with a significant impact on patient outcomes. Teh et al demonstrated that stroke-associated pneumonia affects approximately 11.7% of stroke patients and is associated with significantly increased mortality, prolonged hospitalization, and worse functional outcomes at discharge.^[26]^ Similarly, Katzan et al reported pneumonia in 6.9% of stroke patients, with a nearly threefold increased risk of 30-day mortality.^[27]^ While a previous meta-analysis^[28]^ has identified general risk factors for pneumonia in hospitalized stroke patients, such as malnutrition, stroke severity, and advanced age, few studies have specifically investigated anemia as an independent predisposing factor. Patel et al.^[29]^ reported an association between anemia and increased in-hospital pneumonia risk in patients with acute stroke; however, their analysis did not assess long-term pneumonia outcomes or account for differences in anemia severity. Our study extends these findings by demonstrating a graded association between increasing anemia severity and pneumonia risk across both short- and long-term timeframes in a large, propensity-matched cohort of ischemic stroke patients.

Despite its strengths, our study has several limitations that warrant consideration. First, as this was a retrospective observational study, we could not establish causality between pre-stroke anemia and post-stroke AKI. Although propensity score matching balanced the measured baseline covariates, substantial missingness in albumin, HbA1c, and eGFR may have limited its ability to account fully for metabolic and renal differences between the groups. Residual confounding from incompletely measured and unmeasured factors therefore remains possible. For instance, data on stroke severity, including the National Institutes of Health Stroke Scale and infarct size, were not available, precluding direct adjustment for baseline neurologic deficit. In addition, the uniform hemoglobin threshold used in the primary mixed-sex cohort may have resulted in exposure misclassification in some female patients because diagnostic thresholds for anemia differ by sex. Nevertheless, the sex-specific AKI estimates were similar (HR 1.85 in males and 1.84 in females) when sex-specific hemoglobin thresholds were applied. This consistency suggests that the primary association was not entirely explained by the uniform hemoglobin threshold used in the mixed-sex analysis. Second, our database lacked detailed information on the etiology and chronicity of anemia, which might have influenced its relationship with post-stroke outcomes. Different types of anemia (e.g., iron deficiency, anemia of chronic disease, and hemolytic anemia) may have distinct pathophysiological effects on kidney function after stroke.^[30]^ In addition, patients with hemoglobin <8 g/dL were excluded rather than analyzed as a separate subgroup to maintain cohort homogeneity and reduce potential confounding from acute critical illness or transfusion-related effects. Therefore, our findings are applicable primarily to patients with mild-to-moderate anemia and may not be generalizable to those with more severe anemia. Third, information on specific stroke treatments (e.g., thrombolysis and thrombectomy) and periprocedural factors that might affect renal function (e.g., contrast media volume, hypotension episodes, blood transfusion, and ICU-level management) was underreported across participating health systems, limiting our ability to fully account for treatment-related pathways influencing outcomes. These factors could moderate the relationship between anemia and post-stroke AKI. In addition, AKI was ascertained using diagnostic codes and therefore represents EHR-recorded AKI rather than KDIGO-defined AKI based on serial serum creatinine or urine output measurements. Consequently, milder or uncoded AKI events may have been under-ascertained. This distinction should be considered when comparing our findings with prospective studies using systematic KDIGO-based surveillance. Fourth, recurrent ischemic strokes during follow-up were not treated as censoring events and therefore could have contributed to subsequent AKI risk. In addition, although race was included among the measured covariates used for propensity score matching, formal effect-modification analyses by ethnicity were not performed. Therefore, potential heterogeneity of the observed association across ethnic groups remains uncertain. Fifth, formal Fine–Gray competing-risk analysis could not be performed because this method is not supported by the TriNetX analytic framework. Accordingly, the analysis restricted to patients who survived for at least 1 year should be viewed as a sensitivity analysis for potential competing mortality rather than as a formal competing-risk analysis. Although the associations remained consistent in this analysis, residual competing-risk bias related to mortality cannot be excluded. The proportional hazards assumption was not satisfied for AKI and pneumonia. Accordingly, the reported Cox hazard ratios for these outcomes should be interpreted as summary estimates over the 1-year follow-up period rather than as constant relative hazards over time. Finally, because the study population was derived from healthcare organizations participating in the TriNetX Research Network, the generalizability of our findings to patients treated outside these health systems may be limited. In addition, while our 1-year follow-up period captured most post-stroke renal complications, longer-term consequences of anemia on kidney function beyond this timeframe remain unexplored.

In conclusion, pre-stroke anemia was associated with a higher risk of AKI and other adverse outcomes following ischemic stroke. These findings reflect statistically significant associations observed in an observational analysis and should not be interpreted as evidence of a causal relationship. Accordingly, these results should be considered hypothesis-generating. Future prospective investigations are warranted to confirm these associations, clarify potential underlying biological pathways, and determine whether incorporation of hemoglobin levels into multivariable prognostic models improves risk stratification in patients with ischemic stroke.

## Author contributions

**Conceptualization:** I-Wen Chen, Chia-Li Kao, Yi-Chen Lai, Kuo-Chuan Hung.

**Data curation:** I-Wen Chen, Chia-Li Kao, Yi-Chen Lai, Kuo-Chuan Hung.

**Formal analysis:** I-Wen Chen, Chia-Li Kao, Yi-Chen Lai, Kuo-Chuan Hung.

**Investigation:** Kuo-Chuan Hung.

**Methodology:** I-Wen Chen, Yi-Chen Lai, Kuo-Chuan Hung.

**Software:** I-Wen Chen.

**Writing – original draft:** I-Wen Chen, Kuo-Chuan Hung.

**Writing – review & editing:** I-Wen Chen, Kuo-Chuan Hung.



## References

[1] GBD 2019 Stroke Collaborators. Global, regional, and national burden of stroke and its risk factors, 1990–2019: a systematic analysis for the Global Burden of Disease Study 2019. Lancet Neurol. 2021;20:795–820. 10.1016/S1474-4422(21)00252-0 PMC8443449 34487721

[2] Covic A Schiller A Mardare NG. The impact of acute kidney injury on short-term survival in an Eastern European population with stroke. Nephrol Dial Transplant. 2008;23:2228–34. 10.1093/ndt/gfm591 17989102

[3] Grosjean F Tonani M Maccarrone R. Under-recognized post-stroke acute kidney injury: risk factors and relevance for stroke outcome of a frequent comorbidity. Int Urol Nephrol. 2019;51:1597–604. 10.1007/s11255-019-02203-4 31250340

[4] Huang Y Wan C Wu G. Acute kidney injury after a stroke: a PRISMA-compliant meta-analysis. Brain Behav. 2020;10:e01722. 10.1002/brb3.1722 PMC7507391 32757350

[5] Wang D Guo Y Zhang Y Li Z Li A Luo Y. Epidemiology of acute kidney injury in patients with stroke: a retrospective analysis from the neurology ICU. Intern Emerg Med. 2018;13:17–25. 10.1007/s11739-017-1703-z 28656546

[6] Zorrilla-Vaca A Ziai W Connolly ES Jr Geocadin R Thompson R Rivera-Lara L. Acute kidney injury following acute ischemic stroke and intracerebral hemorrhage: a meta-analysis of prevalence rate and mortality risk. Cerebrovasc Dis. 2018;45:1–9. 10.1159/000479338 29176313

[7] Khatri M Himmelfarb J Adams D Becker K Longstreth WT Tirschwell DL. Acute kidney injury is associated with increased hospital mortality after stroke. J Stroke Cerebrovasc Dis. 2014;23:25–30. 10.1016/j.jstrokecerebrovasdis.2012.06.005 PMC3507321 22818389

[8] Arnold J Ng KP Sims D Gill P Cockwell P Ferro C. Incidence and impact on outcomes of acute kidney injury after a stroke: a systematic review and meta-analysis. BMC Nephrol. 2018;19:283. 10.1186/s12882-018-1085-0 PMC6196566 30348107

[9] Chun S Han K Kim B. Elevated risk of end-stage kidney disease in stroke patients: a population-based observational study. Int J Stroke. 2024;20:461–70. 10.1177/17474930241295890 39410665

[10] Wu CL Tsai CC Kor CT. Stroke and risks of development and progression of kidney diseases and end-stage renal disease: a nationwide population-based cohort study. PLoS One. 2016;11:e0158533. 10.1371/journal.pone.0158533 PMC4927175 27355475

[11] Yoo J Hong JH Lee SJ. Acute kidney injury after endovascular treatment in patients with acute ischemic stroke. J Clin Med. 2020;9:1471. 10.3390/jcm9051471 PMC7291207 32422921

[12] Verwijmeren L Bosma M Vernooij LM. Associations between preoperative biomarkers and cardiac surgery-associated acute kidney injury in elderly patients: a cohort study. Anesth Analg. 2021;133:570–7. 10.1213/ANE.0000000000005650 34153017

[13] DeCarlo C Mohebali J Dua A Conrad MF Mohapatra A. Preoperative anemia is associated with postoperative renal failure after elective open aortic repair. J Surg Res. 2023;291:187–94. 10.1016/j.jss.2023.05.033 37442045

[14] Oprea AD Del Rio JM Cooter M. Pre- and postoperative anemia, acute kidney injury, and mortality after coronary artery bypass grafting surgery: a retrospective observational study. Can J Anaesth. 2018;65:46–59. 10.1007/s12630-017-0991-0 29098634

[15] Walsh M Garg AX Devereaux P Argalious M Honar H Sessler DI. The association between perioperative hemoglobin and acute kidney injury in patients having noncardiac surgery. Anesth Analg. 2013;117:924–31. 10.1213/ANE.0b013e3182a1ec84 24023017

[16] Shema-Didi L Ore L Geron R Kristal B. Is anemia at hospital admission associated with in-hospital acute kidney injury occurrence? Nephron Clin Pract. 2010;115:c168–76. 10.1159/000312881 20407277

[17] Han SS Baek SH Ahn SY. Anemia is a risk factor for acute kidney injury and long-term mortality in critically ill patients. Tohoku J Exp Med. 2015;237:287–95. 10.1620/tjem.237.287 26607258

[18] Ludwig RJ Anson M Zirpel H. A comprehensive review of methodologies and application to use the real-world data and analytics platform TriNetX. Front Pharmacol. 2025;16:1516126. 10.3389/fphar.2025.1516126 PMC11931024 40129946

[19] Chen IW Yu TS Lai YC Chang YJ Hung KC. Association between iron deficiency anemia and risk of hearing loss among female patients: a multi-institutional cohort study. Front Nutr. 2025;12:1718261. 10.3389/fnut.2025.1718261 PMC12728430 41450550

[20] Hung KC Yu TS Lai YC. Perioperative ketamine exposure and postoperative atrial fibrillation/flutter risk after video-assisted thoracoscopic surgery: a multi-institutional study. Drug Des Devel Ther. 2025;19:11173–85. 10.2147/DDDT.S569143 PMC12717812 41425499

[21] Hung KC Yu TS Lai YC. Vitamin D deficiency and subsequent risk of obstructive sleep apnea: a multi-institutional retrospective study. Front Nutr. 2025;12:1651712. 10.3389/fnut.2025.1651712 PMC12648596 41311801

[22] Lin YL Wei YC Chao CH Weng WC Huang WY. Association between hemoglobin level and clinical outcomes in ischemic stroke patients with high-grade carotid artery stenosis. Clin Neurol Neurosurg. 2025;250:108793. 10.1016/j.clineuro.2025.108793 40010241

[23] Barlas RS Honney K Loke YK. Impact of hemoglobin levels and anemia on mortality in acute stroke: analysis of UK regional registry data, systematic review, and metaanalysis. J Am Heart Assoc. 2016;5:e003019. 10.1161/JAHA.115.003019 PMC5015269 27534421

[24] Milionis H Papavasileiou V Eskandari A D’Ambrogio-Remillard S Ntaios G Michel P. Anemia on admission predicts short-and long-term outcomes in patients with acute ischemic stroke. Int J Stroke. 2015;10:224–30. 10.1111/ijs.12397 25427291

[25] Zhang R Xu Q Wang A. Hemoglobin concentration and clinical outcomes after acute ischemic stroke or transient ischemic attack. J Am Heart Assoc. 2021;10:e022547. 10.1161/JAHA.121.022547 PMC9075388 34845923

[26] Teh WH Smith CJ Barlas RS. Impact of strokeassociated pneumonia on mortality, length of hospitalization, and functional outcome. Acta Neurol Scand. 2018;138:293–300. 10.1111/ane.12956 29749062

[27] Katzan IL Cebul RD Husak SH Dawson NV Baker DW. The effect of pneumonia on mortality among patients hospitalized for acute stroke. Neurology. 2003;60:620–5. 10.1212/01.wnl.0000046586.38284.60 12601102

[28] Guo T Dou L Zhou X. Risk factors of stroke complicated with hospital-acquired pneumonia: a systematic review and meta-analysis of cohort studies. Ann Palliat Med. 2021;10:12381–9. 10.21037/apm-21-3278 35016483

[29] Patel UK Kodumuri N Dave M. Stroke-associated pneumonia: a retrospective study of risk factors and outcomes. Neurologist. 2020;25:39–48. 10.1097/NRL.0000000000000269 32358460

[30] Barlas RS McCall SJ Bettencourt-Silva JH. Impact of anaemia on acute stroke outcomes depends on the type of anaemia: evidence from a UK stroke register. J Neurol Sci. 2017;383:26–30. 10.1016/j.jns.2017.09.047 29246615

