# Supplementary material for: Association between pre-stroke anemia and risk of acute kidney injury following ischemic stroke: A multi-institutional retrospective cohort study
Source: Medicine (Baltimore). 2026 Nov 6;105(45):e51449. doi: 10.1097/MD.0000000000051449 (PMC13747610; doi:10.1097/MD.0000000000051449)
Supplement: Supplementary file 1 [file medi-105-e51449-s001.docx]

**Supplemental Table S1.** Availability and missingness of laboratory variables in the propensity score-matched cohorts

| Laboratory variable | Anemia group available, n (%) | Anemia group missing, n (%) | Control group available, n (%) | Control group missing, n (%) |
| --- | --- | --- | --- | --- |
| Albumin | 25,251 (40.8%) | 36,645 (59.2%) | 22,519 (36.4%) | 39,377 (63.6%) |
| Hemoglobin A1c | 14,388 (23.2%) | 47,508 (76.8%) | 14,194 (22.9%) | 47,702 (77.1%) |
| eGFR | 23,690 (38.3%) | 38,206 (61.7%) | 21,682 (35.0%) | 40,214 (65.0%) |

Missingness was calculated as the matched cohort size minus the number of patients with an available laboratory measurement. No imputation was performed for missing laboratory values.
